# Entomological surveillance of invasive *Aedes* mosquitoes in Mazandaran Province, northern Iran from 2014 to 2020

**DOI:** 10.1038/s41598-023-35860-8

**Published:** 2023-05-29

**Authors:** Seyed Hassan Nikookar, Ali Charkame, Asghar Nezammahalleh, Eslam Moradi-Asl, Ahmadali Enayati, Mahmoud Fazeli-Dinan, Mohammad Mehdi Sedaghat, Morteza Zaim

**Affiliations:** 1 Health Sciences Research Center, Department of Medical Entomology and Vector Control, School of Public Health, Mazandaran University of Medical Sciences, Sari, Iran; 2 Present Address: Medical Entomology, Health Expert of the Health Deputy, Mazandaran University of Medical Sciences, Sari, Iran; 3 Department of Public Health, School of Public Health, Ardabil University of Medical Sciences, Ardabil, Iran; 4 Head of Medical Entomology Department, School of Public Health and Health Sciences Research Center, Mazandaran University of Medical Sciences, Sari, Iran; 5 Department of Medical Entomology and Vector Control, School of Public Health, Tehran University of Medical Sciences, Tehran, Iran; 6 Affiliated Professor, Department of Medical Entomology and Vector Control, School of Public Health, Tehran University of Medical Sciences, Tehran, Iran

**Keywords:** Environmental sciences, Diseases, Health care, Ecology, Ecology, Invasive species

## Abstract

Mosquitoes are the most important vectors of serious infectious diseases in the world. Dengue, Zika, chikungunya and yellow fever are emerging and re-emerging infectious diseases, associated with the distribution of two key vectors i.e. *Aedes aegypti* and *Aedes albopictus* throughout the world including countries neighbouring Iran. Entomological surveillance was planned and performed monthly from May to December during 2014–2020 in selected counties of Mazandaran Province, North of Iran, by ovitrap, larval collection, hand catch and human baited trap. Overall, 4410 *Aedes* specimens including 2376 larvae (53.9%) and 2034 (46.1%) adults belonging to six species, namely *Aedes vexans, Aedes geniculatus, Aedes caspius, Aedes echinus, Aedes pulcritarsis* and *Aedes flavescence* were collected and morphologically identified. Over the seven years of surveillance, *Ae. aegypti* and *Ae. albopictus* were not found by any sampling method. *Aedes vexans* and *Ae*. *geniculatus* were the most abundant species, their populations peaked in October and November and was positively correlated with precipitation and relative humidity. *Aedes flavescence* was a new species record for the province. A flowchart for planning and implementation of invasive mosquito surveillance for provincial health authorities in the country is proposed. These surveillance efforts provide basic and timely information for the health system to act promptly on integrated and intensified surveillance and control programs should *Ae. aegypti* and *Ae. albopictus* detected in the province.

## Introduction

Mosquitoes are the most important vectors of arthropod-borne diseases in the world as they transmit malaria and arboviral diseases^1^. Globalization coupled with changes in ecosystems and climate, as well as mosquitoes' capacity to adapt to a changing environment, support the emergence and re-emergence of mosquito-borne diseases and potential for the establishment of invasive vector species^2^.

Dengue fever, Zika and chikungunya, transmitted by *Aedes aegypti* and *Aedes albopictus,* are on the rise globally probably linked to poor control as well as the lack of efficient antivirals or vaccines^3^. *Aedes aegypti* is known as the yellow fever mosquito, originated from sub-Saharan Africa and evolved from a wild and zoophilic ancestral species, *Ae. aegypti formosus*^4^. The species is established in many tropical and subtropical regions due to international trade, globalization and human activities from the 15th through twentieth century^5^. It is the most anthropophilic mosquito, prefers human settlements for resting place and blood source availability^6^ . The species feeds preferably on humans, does so several times per gonotrophic cycle, is most active during daytime with peak biting activity at dawn and dusk, and typically rests indoors, biology and behavior that facilitates its potential as an efficient vector of arboviruses in human-mosquito cycles^7^.

*Aedes albopictus* is known as the Asian tiger mosquito or forest mosquito, native to Southeast Asia, spread to islands in the Indian and Pacific Oceans. The species is regarded as the most invasive mosquito in the world^3^, now being established in North, Central and South America, Africa, Oceania, southern Europe and the Middle East. Similar to *Ae. aegypti*, it is also a daytime-biting mosquito that is most active during the morning and evening^8^. It is an opportunistic species that feeds on wide ranges of hosts and tends to rest outdoors^9^, but it is, more recently, displaying intense anthropophilic behavior like *Ae. aegypti*^3^.

Iran is at risk of *Aedes*-borne diseases because of the presence of *Ae. albopictus* and or *Ae. aegypti* in neighbouring countries including Afghanistan, Armenia, Oman, Pakistan, Saudi Arabia, Turkey and Yemen and also the epidemics and outbreaks of dengue fever and chikungunya infections in Pakistan, Saudi Arabia, Yemen and Oman^10,11^. Although there are no reports of Zika virus in the WHO Eastern Mediterranean Region, but risk of autochthonous transmission of Zika in areas of the Red Sea coast and Pakistan, following the introduction from endemic countries, cannot be ignored^12^.

In Iran, *Ae. albopictus* and *Ae. aegypti* have been sighted in south-eastern and south of Iran in recent years^11^, revelations that are of concern for the country as it may pave the way for their further distribution and local transmission of the diseases from imported cases. The first imported case of dengue fever was documented in 2008 in Iran^13^. In 2013, 15 positive cases of dengue fever from Sistan and Baluchestan and Kurdistan Provinces were reported, eight of whom had travelled to Malaysia, India and Thailand and seven cases were individuals with no clear travel history abroad^14^. In 2019, 50 imported cases of dengue and 53 imported cases of chikungunya were identified in Iran^11^. Therefore, entomological, laboratory and human surveillance are crucial for the prevention and control of the arboviral diseases.

Surveillance is considered as the cornerstone of integrated mosquito management program (IMM), and is a set of methods performed in response to known risk of mosquito-borne diseases for the possibility of informed decision making^15^. Regarding the importance of mosquito surveillance, it has been emphasized that each epidemic early in its development period can be prevented or reduced by precise vector surveillance and control^15^.

Mazandaran Province has suitable climate conditions for the development and diversity of vectors^16,17^. It connects with the neighboring country Russia in the North, where there are reports of the presence of *Ae. aegypti* and *Ae. albopictus*^18^, through three active ports (Noshahr, Fereydunkenar and Amirabad). The ports eventually are connected through the Volga-Don Canal to the Black Sea region^19^. The province also has one international airport with flights from infested countries including Saudi Arabia, located in Sari County, the capital of the province. Therefore, Mazandaran Province is prone to the risk of importation of invasive *Aedes* species. In these situations, entomological surveillance especially at the ports of entries is the key for early detection of the occurrence, establishment and abundance of invasive *Aedes* vectors and provides baseline data, develops the capability and capacity of ports and airports health officers and health center personnel for implementing timely preventive measures when and where necessary.

Therefore, considering the concern about the possible entry of these species from the northern neighbouring or other countries as well as the geographical and ecological suitability of Mazandaran Province, this study was conducted with the aim of (1) establishing the initial entomological surveillance of invasive *Aedes* species i.e. *Ae. aegypti* and *Ae. albopictus* in line with the national guidelines, (2) designing a flowchart for the entomological surveillance program, (3) providing a dataset for *Aedes* fauna in northern Iran, (4) investigating the effects of climate variables on *Aedes* population fluctuations, (5) Mapping of historical and contemporary distribution records of *Ae. aegypti* and *Ae. albopictus* in Iran.

## Result

### Flowchart design for entomological surveillance program

**Figure 1:**
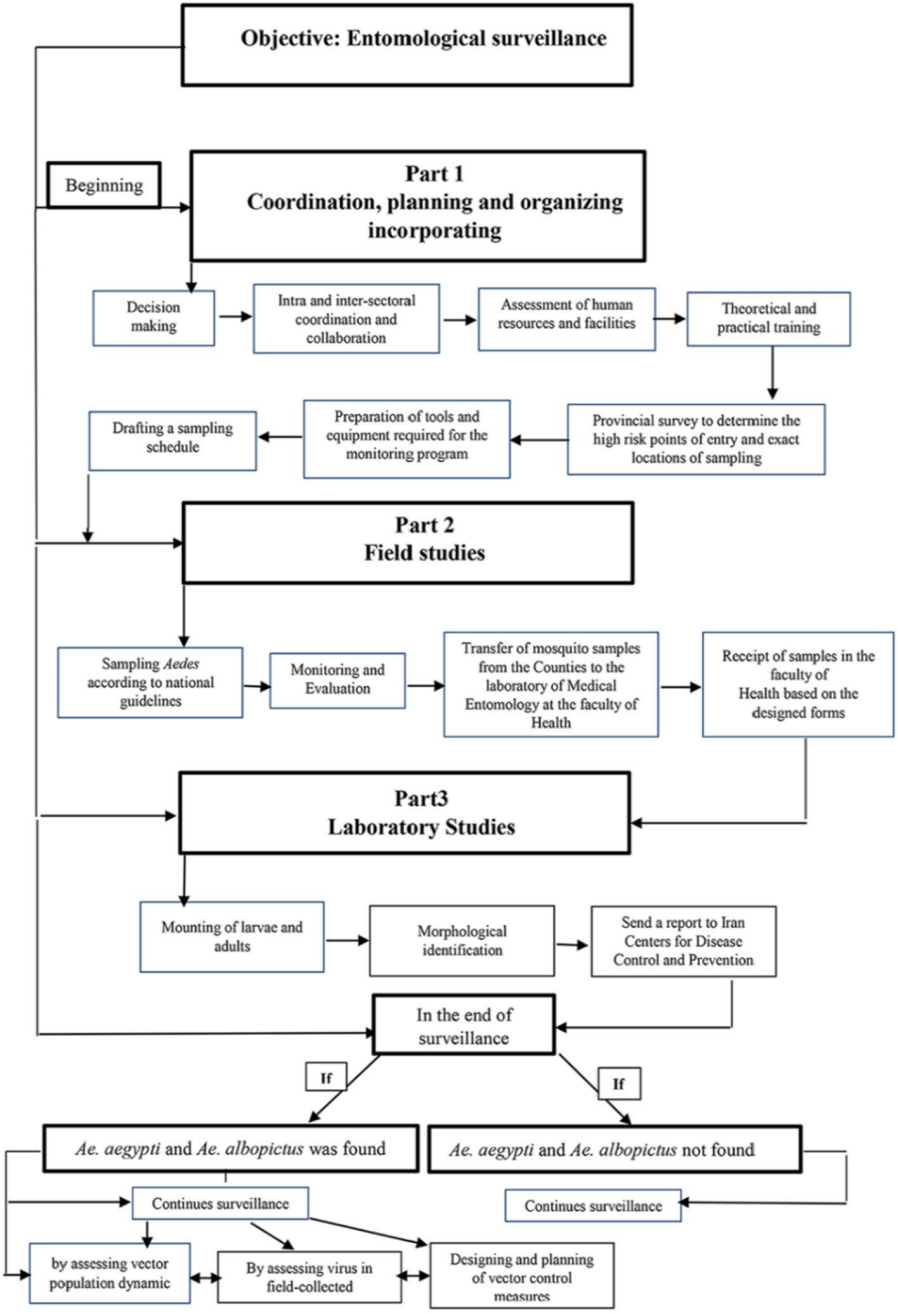
Flowchart of operational processes in entomological surveillance program of invasive *Aedes* vectors in Mazandaran Province, northern Iran.

A flowchart was designed in three categories of management, field and laboratory works in the present study to systematically improve the coherence of the sampling process of invasive *Aedes* and to ensure adequate quality of the study (Fig. 1).

## *Aedes* mosquitoes sampling

**Figure 2:**
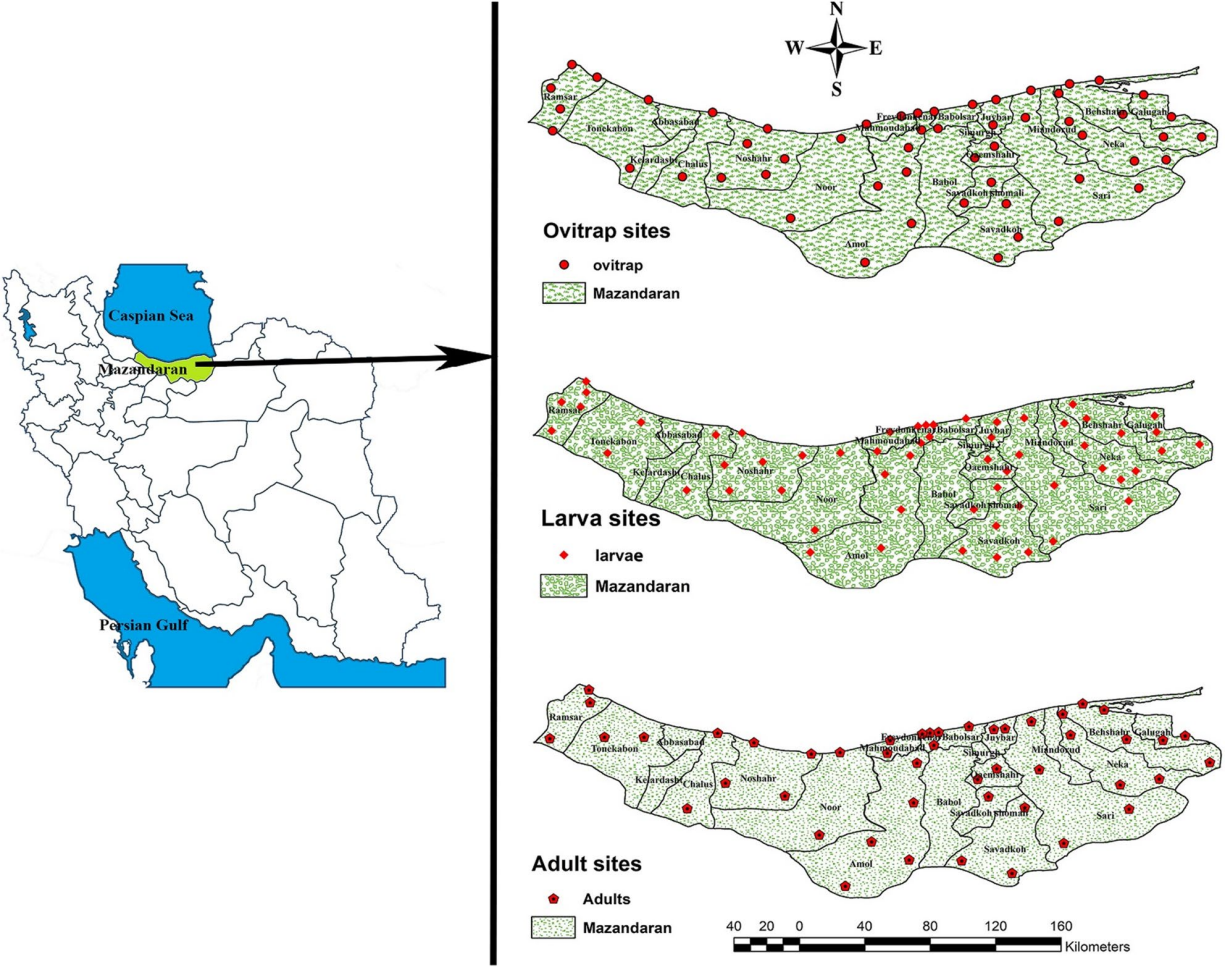
Map of the study area along with sampling sites (ovitrap, larvae and adults) of mosquitoes in Mazandaran Province, northern Iran during the surveillance period, 2014–2020 (ArcMap GIS10.8 and Adobe Photoshop 2021 v22.4.1.211 were used to draw the graphs).

A total of 4410 *Aedes* specimens including 2376 larvae (53.9%) and 2034 (46.1%) adults belonging to 6 species, namely *Ae. vexans, Ae. geniculatus, Ae. caspius, Ae. echinus, Ae. pulchitarsis* and *Ae. flavescence* were collected from Mazandaran Province and identified using morphological characteristics. Among these, *Ae. flavescence* was a new record for Mazandaran Province. No specimens of *Ae. aegypti* and *Ae. albopictus* were found during the monitoring program from 2014 to 2020 (Table 1). Although the highest number of *Aedes* mosquitoes were caught in 2017 (22%), the maximum and minimum species diversity was recorded in 2015 (with 6 species richness) and 2014 (with 2 species richness), respectively. The highest number of *Aedes* specimens was collected by larval sampling method in the study area (Table 1). Interestingly, no specimens were collected by ovitraps and inspection in ships.(Fig. 2)

**Table 1:** Numbers and percentage of *Aedes* specimens by different collection methods in Mazandaran Province, northern Iran during the entomological surveillance period, 2014–2020.

Collection date	Species	County	Larvae			adult		No. Species	Total (larvae and adults)		Larvae collection	Daily biting	Hand catch
			N		%	N	%		N	%			
2014	*Ae. vexans*	Ma, No, Nos	–		–	1270	97.09	2	1308	29.6		+	
	*Ae. caspius*	Ga, Ne, Nos	–		–	38	2.91					+	
2015	*Ae. vexans*	Be, Sa, Sav, Am, Nos, Ra	117		33.43	84	41.38	6	553	12.54	+	+	+
	*Ae. echinus*	Nos	2		0.57	–	–				+		
	*Ae. geniculatus*	Be, Ne,Sa, Sav, Am, Nos, Ra		231	66	114	56.16				+	+	
	*Ae. flavescence*	Nos	–		–	1	0.49					+	
	*Ae. pulcritarsis*	Ne	–		–	1	0.49					+	
	*Ae. caspius*	Sa	–		–	3	1.47					+	
2016	*Ae. vexans*	Be, Ne,Sa, Sav, Am, Fe, Nos, Ra	250		52.08	110	39.29	4	760	17.23	+	+	
	*Ae. geniculatus*	Be, Ne,Sa, Sav, Am, Fe, Nos, Ra	230		47.92	114	40.71				+	+	
	*Ae. pulcritarsis*	Ne	–		–	6	2.14					+	
	*Ae. caspius*	Be, Ne, Nos,	–		–	50	17.86					+	
2017	*Ae. vexans*	Be, Ne, Am, Nos	470		55.69	86	68.25	3	970	22	+	+	
	*Ae. geniculatus*	Be, Ne, Sa, Sav, Am, Nos	360		42.65	40	31.75				+	+	
	*Ae. caspius*	Be, Ne, Nos	14		1.66	–	–				+		
2018	*Ae. vexans*	Beh, Nos	100		69.44	–	–	3	144	3.26	+		
	*Ae. caspius*	Nos	2		1.39	–	–				+		
	*Ae. geniculatus*	Be, Nos	42		29.17	–	–				+		
2019	*Ae. vexans*	Be, Nos	58		70.73	–	–	3	82	1.86	+		
	*Ae. caspius*	Nos	1		1.22	–	–				+		
	*Ae. geniculatus*	Beh, Nos	23		28.05	–	–				+		
2020	*Ae. vexans*	Be, Ne, Am, Nos	76		15.97	50	42.74	4	593	13.45	+	+	
	*Ae. geniculatus*	Be, Ne, Sa, Sav, Am, Nos	390		81.93	40	34.19				+	+	
	*Ae. pulcritarsis*	Be, Ne	–		–	7	5.98					+	
	*Ae. caspius*	Be, Ne, Sa, Sav, Nos, Ra	10		2.10	20	17.09				+	+	
Total			2376		53.9	2034	46.1	–	4410	100			

The first two or three letters of the Counties names were used in this table. These include: Galugah County (Ga), Behshahr (Be), Neka (Ne), Sari (Sa), Ghaemshahr (Gh), Savadkooh (Sav), Fereydunkenar (Fe), Amol (Am), Mahmudabad (Ma), Noor (No), Noshahr (Nos)and Ramsar (Ra).

### Spatio-temporal distribution of the *Aedes* species during the study period

**Figure 3:**
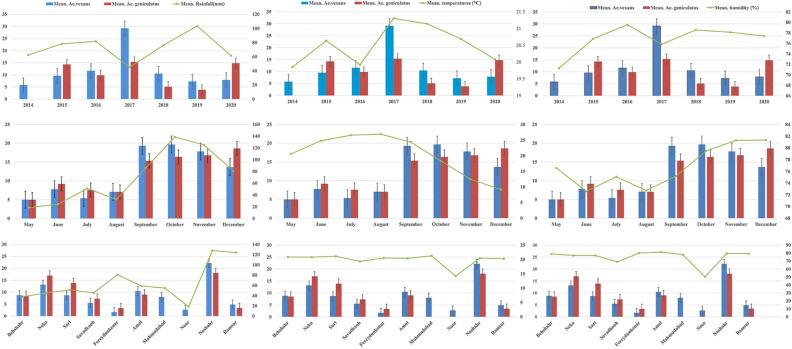
Comparison of the mean population density of the most common species, *Ae vexans and Ae geniculatus* by month, year, and county along with impact of meteorological variables in Mazandaran Province, northern Iran, during the surveillance period, 2014–2020.

Means and standard deviations of *Aedes* populations were calculated based on year, month and counties in the province. The non-parametric Kruskal–Wallis test showed that the population abundance of *Ae. vexans* and *Ae. geniculatus* changed significantly by year, month, and county over the study period (Table 2). These species showed the most significant differences in 2017 (29.16 ± 31.167; 15.38 ± 14.770) and Noshahr County (22.16 ± 25.836; 18.04 ± 16.216) compared to other years and counties (Table 2 and Fig. 3). *Aedes vexans* was collected with the maximum mean abundance in October (19.68 ± 25.604) and *Ae. geniculatus* in December (18.60 ± 16.008), which was significantly different from other months (Table 2 and Fig. 3). There was no significant difference between the population abundance of other *Aedes* species by year, month, and county during the monitoring period in the area (Table 2).

**Table 2:** Mean and standard deviation of *Aedes* mosquitoes (summed larvae and adults), along with the number of occurrences, by year, month, and county collected during the surveillance in Mazandaran Province, northern Iran from 2014–2020.

Species	Year/Month/County	No. of occurrence	No. of specimen	Mean	Std. deviation	95% Confidence interval for mean	95% Confidence interval for mean	*P* value
						Lower bound	Upper bound	
*Ae.vexans*	2014	10	58	5.80	6.512	1.14	10.46	0.008
	2015	21	201	9.57	10.332	4.87	14.27	
	2016	31	360	11.61	11.029	7.57	15.66	
	2017	19	554	29.16	31.167	14.14	44.18	
	2018	10	105	10.50	7.382	5.22	15.78	
	2019	8	58	7.25	4.132	3.80	10.70	
	2020	16	126	7.88	4.272	5.60	10.15	
	Total	115	1462	12.71	16.506	9.66	15.76	
*Ae.geniculatus*	2014	–	–					0.004
	2015	31	443	14.29	13.059	9.50	19.08	
	2016	32	316	9.88	9.189	6.56	13.19	
	2017	26	400	15.38	14.770	9.42	21.35	
	2018	8	41	5.13	4.324	1.51	8.74	
	2019	6	23	3.83	2.639	1.06	6.60	
	2020	29	430	14.83	13.387	9.74	19.92	
	Total	132	1653	12.52	12.341	10.40	14.65	
*Ae. caspius*	2014	8	38	4.75	5.09	.49	9.01	0.198
	2015	1	3	3.00				
	2016	9	50	5.56	4.927	1.77	9.34	
	2017	5	14	2.80	2.04	0.26	5.34	
	2018	2	2	1.00	.000	1.00	1.00	
	2019	1	1	1.00				
	2020	12	30	2.50	2.468	0.93	4.07	
	Total	38	138	3.63	3.83	2.37	4.89	
*Ae. pulchritarsis*	2014	–	–					0.761
	2015	–	–					
	2016	2	6	3.00	2.828	− 22.41	28.41	
	2017	–	–					
	2018	–	–					
	2019	–	–					
	2020	3	7	2.33	1.155	− 0.54	5.20	
	Total	5	13	2.60	1.673	0.52	4.68	
*Ae. vexans*	May	3	15	5.00	6.083	− 10.11	20.11	0.001
	June	14	109	7.79	5.860	4.40	11.17	
	July	23	124	5.39	4.449	3.47	7.32	
	August	13	92	7.08	4.406	4.41	9.74	
	September	13	251	19.31	22.903	5.47	33.15	
	October	19	374	19.68	25.604	7.34	32.02	
	November	21	374	17.81	17.096	10.03	25.59	
	December	9	123	13.67	14.671	2.39	24.94	
	Total	115	1462	12.71	16.506	9.66	15.76	
*Ae. geniculatus*	May	1	5	5.00				0.002
	June	11	101	9.18	7.195	4.35	14.02	
	July	26	197	7.58	6.646	4.89	10.26	
	August	22	155	7.05	6.090	4.35	9.75	
	September	15	230	15.33	14.085	7.53	23.13	
	October	23	376	16.35	11.625	11.32	21.37	
	November	24	403	16.79	17.139	9.55	24.03	
	December	10	186	18.60	16.008	7.15	30.05	
	Total	132	1653	12.52	12.341	10.40	14.65	
*Ae. caspius*	May	1	5	5.00				0.938
	June	3	11	3.67	3.786	− 5.74	13.07	
	July	4	14	3.50	4.359	− 3.44	10.44	
	August	2	7	3.50	2.121	− 15.56	22.56	
	September	3	13	4.33	4.933	− 7.92	16.59	
	October	12	43	3.58	4.144	0.95	6.22	
	November	11	42	3.82	4.490	0.69	6.31	
	December	2	3	1.00	1.000	− 1.48	3.48	
	Total	38	138	3.63	3.830	2.37	4.89	
*Ae. pulchritarsis*	October	3	5	1.67	1.155	− 1.20	4.54	0.128
	November	2	8	4.00	1.414	− 8.71	16.71	
	Total	5	13	2.60	1.673	0.52	4.68	
*Ae. vexans*	Behshahr	15	132	8.80	7.380	4.71	12.89	0.001
	Neka	23	303	13.17	13.862	7.18	19.17	
	Sari	10	87	8.70	5.716	4.61	12.79	
	Savadkooh	4	22	5.50	4.123	− 1.06	12.06	
	Fereydunkenar	3	5	1.67	1.155	− 1.20	4.54	
	Amol	13	136	10.46	7.423	5.98	14.95	
	Mahmudabad	6	48	8.00	7.746	− 0.13	16.13	
	Noor	3	8	2.67	2.082	− 2.50	7.84	
	Noshahr	31	687	22.16	25.836	12.68	31.64	
	Ramsar	7	34	4.86	3.024	2.06	7.65	
	Total	115	1462	12.71	16.506	9.66	15.76	
*Ae. geniculatus*	Behshahr	20	169	8.45	6.395	5.46	11.44	0.001
	Neka	28	475	16.96	15.495	10.96	22.97	
	Sari	16	222	13.88	9.612	8.75	19.00	
	Savadkooh	14	102	7.29	4.196	4.86	9.71	
	Fereydunkenar	3	10	3.33	1.528	− 0.46	7.13	
	Amol	19	171	9.00	8.048	5.12	12.88	
	Noshahr	27	487	18.04	16.216	11.62	24.45	
	Ramsar	5	17	3.40	3.362	− 0.77	7.57	
	Total	132	1653	12.52	12.341	10.40	14.65	
*Ae. caspius*	Galugah	6	35	5.83	5.529	.03	11.64	0.537
	Behshahr	6	11	1.83	0.753	1.04	2.62	
	Neka	10	27	2.70	2.710	0.76	4.64	
	Sari	1	2	2.00				
	Savadkooh	3	7	2.33	1.155	− 0.54	5.20	
	Noshahr	10	53	5.30	4.900	1.79	8.81	
	Ramsar	2	3	1.50	0.707	− 4.85	7.85	
	Total	38	138	3.63	3.830	2.37	4.89	
*Ae. pulchritarsis*	Behshahr	1	3	3.00				0.709
	Neka	4	10	2.50	1.915	− .55	5.55	
	Total	5	13	2.60	1.673	.52	4.68	

**Figure 4:**
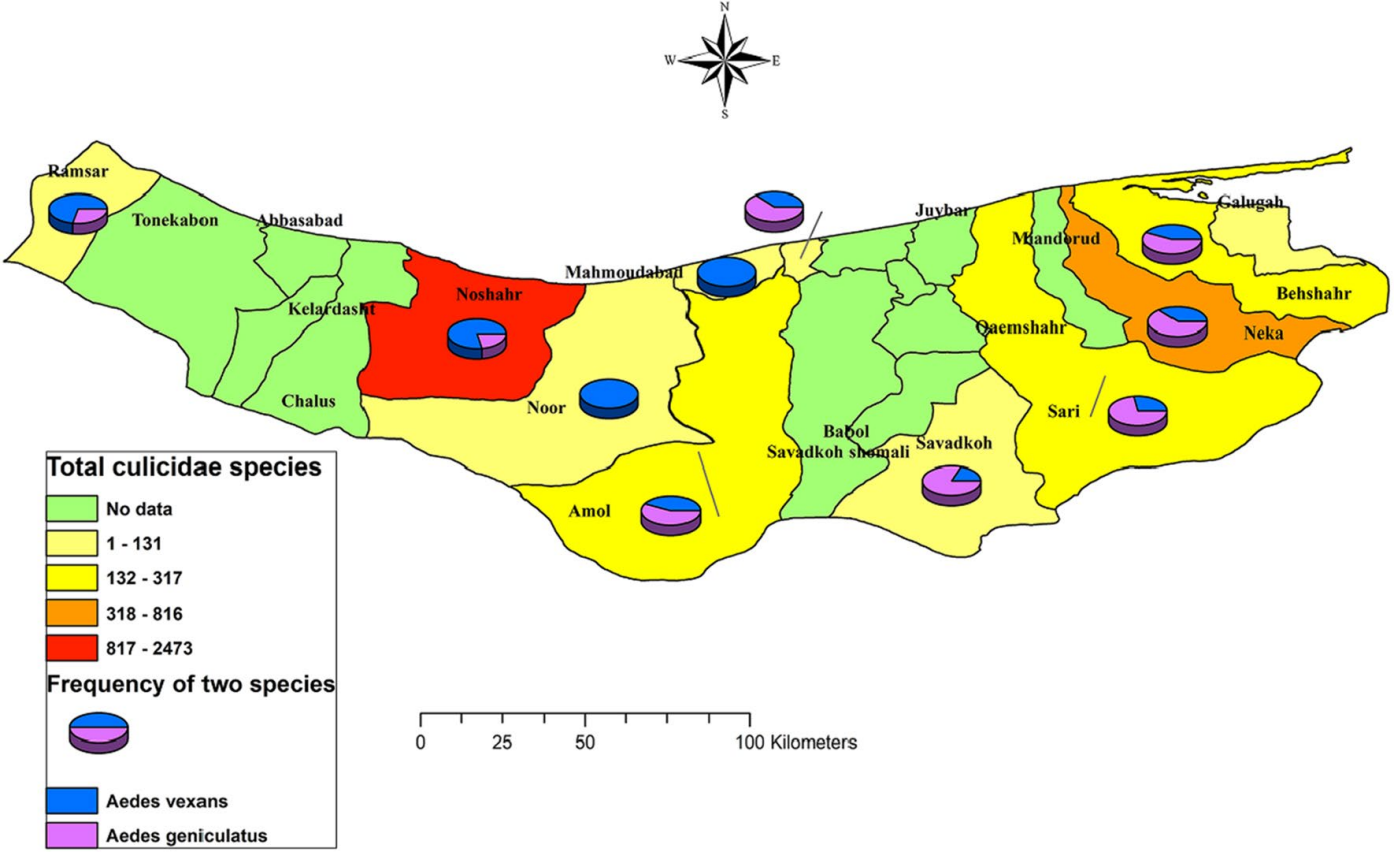
Spatial distribution and frequency of *Ae. vexans* and *Ae. geniculatus****,*** as the most common *Aedes* in Mazandaran Province, northern Iran during the surveillance period, 2014–2020.

Spatial analysis was performed for thirteen counties where *Aedes* species were collected. Most *Aedes* samples were collected in Noshahr and Neka Counties, the former in the west and the latter in the east of Mazandaran Province. Two species of *Ae. vexans* and *Ae. geniculatus* had the highest frequency in the studied counties. The differences between the frequencies of *Ae. geniculatus* in eastern areas and the frequency of *A. vexans* in western areas were significant (Fig. 4).

### Effects of climate variables on the abundance of *Aedes* species

**Figure 5:**
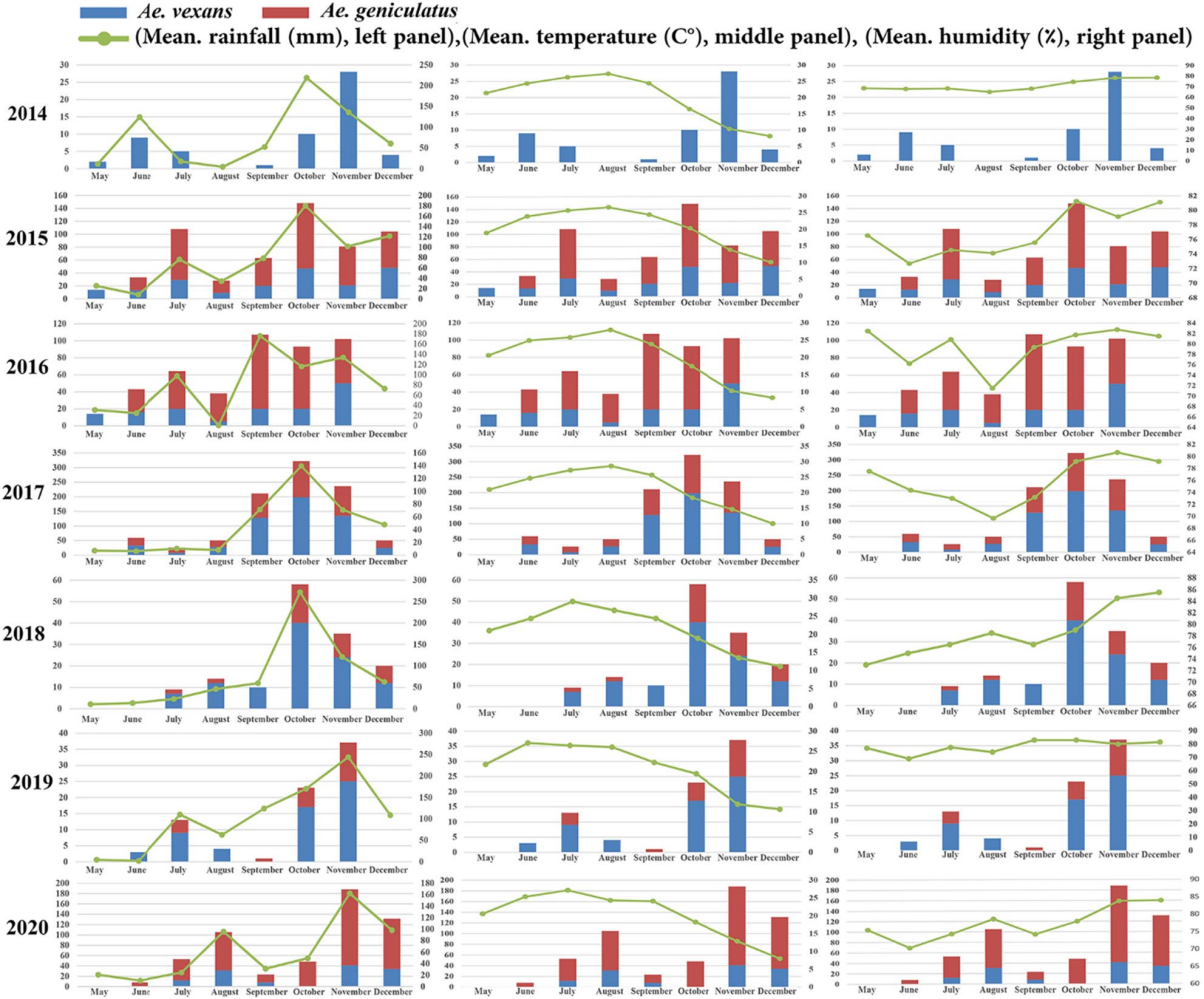
Relation between monthly population fluctuations of most abundant *Aedes* species and meteorological variables in Mazandaran Province, northern Iran.

The meteorological variables (mean rainfall, temperature and humidity) affected the population dynamics of *Aedes* species by month, year and county. Generally, the density of *Ae. vexans* and *Ae. geniculatus* was associated with the meteorological factors i.e. the highest mean humidity (81.35%), temperature (21.3 °C) and rainfall (180 mm) as shown in Fig. 3. Detailed relationship between the monthly meteorological factors and the density of these species during the study period is depicted in Fig. 5. Spearman correlation analysis of *Aedes* population abundance showed that *Ae. vexans* and *Ae geniculatus* had a significant positive correlation with the mean rainfall (r = 0.474; r = 0.374 and *P* < 0.001) and humidity (r = 0.360; r = 0.253 and *P* < 0.001), respectively. These two species were negatively associated with mean temperature with rank correlations of − 0.309 (p = 0.001) and − 0.365 (*P* < 0.001). Results were not significant for other *Aedes* species as shown in Table 3.

**Table 3:** Spearman correlation coefficient between abundance of *Aedes* species (summed larvae and adults) and meteriological variables in Mazandaran Province, northern Iran during the 7-year monitoring, 2014–2020.

Species	Mean rainfall		Mean temperature		Mean humidity	
	Correlation coefficient	Sig	Correlation coefficient	Sig	Correlation coefficient	Sig
*Ae. vexans*	0.474	< 0.001	− 0.309	0.001	.360	< 0.001
*Ae. caspius*	0.179	0.268	0.137	0.399	− 0.080	0.623
*Ae. geniculatus*	0.374	< 0.001	− 0.365	< 0.001	0.253	< 0.001
*Ae. echinus*						
*Ae. flavescence*						
*Ae. pulchritarsis*	0.369	0.541	− 0.527	0.361	0.580	0.306

**Figure 6:**
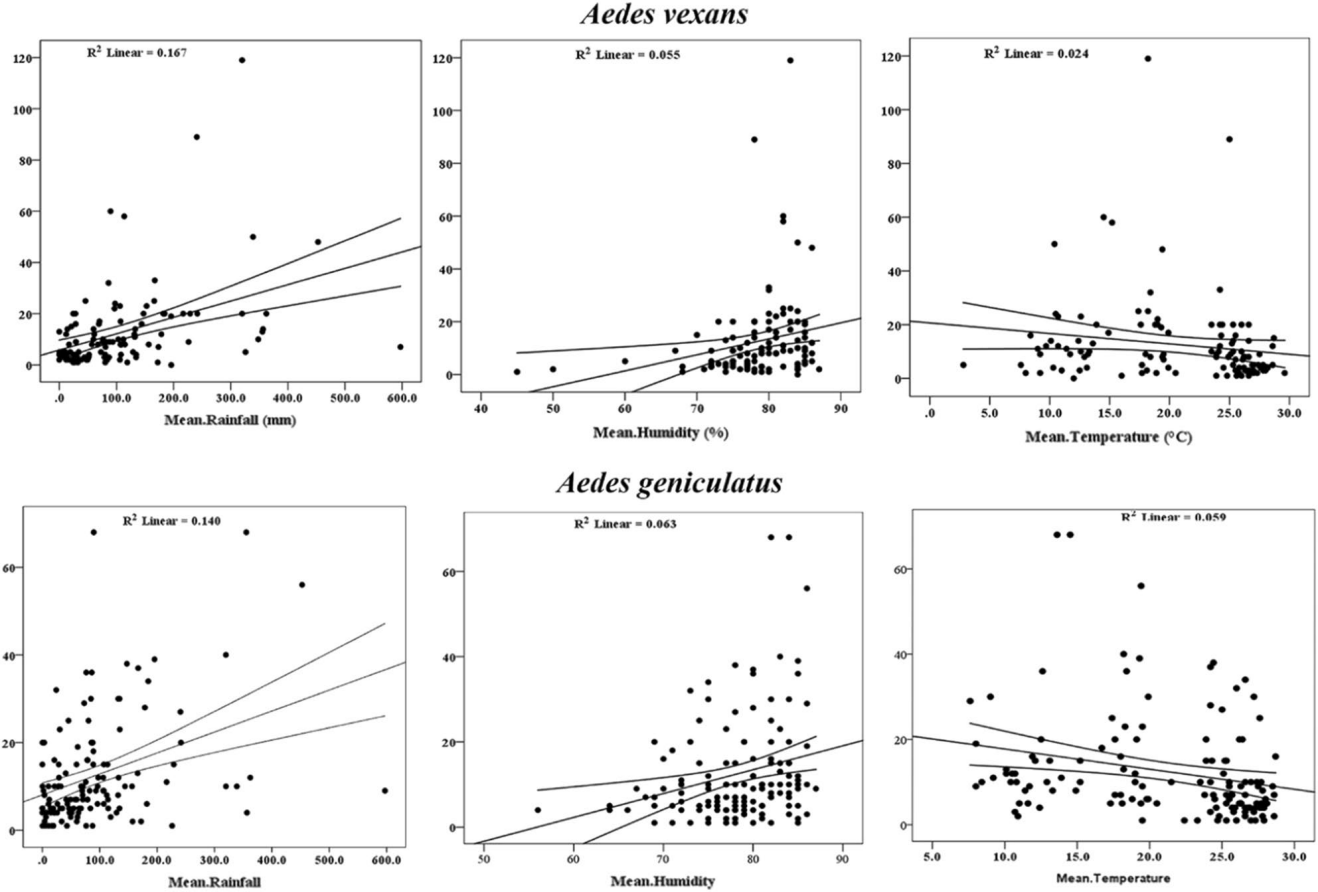
Linear regression model between *Ae. vexans* and *Ae. geniculatus* and the meteriological variables in Mazandaran Province, northern Iran during the surveillance period, 2014–2020.

The regression coefficient (R^2^) was calculated between *Ae vexans* and *Ae. geniculatus* populations, and mean rainfall, humidity, and temperature. The results showed negligible values of 0.167, 0.055 and 0.024 for *Ae. vexans*, and 0.14, 0.063 and 0.059 for *Ae. geniculatus* populations respectively (Fig. 6).

### The trend of monthly population variations of *Ae. vexans* and *Ae. geniculatus* from 2014–2020

Apart from 2014, in nearly all other years of the study period, *Ae. vexans* and *Ae. geniculatus* were collected from the study areas. Only in 2015, both *Ae. vexans* and *Ae. geniculatus* were collected from May to December. However, heterogeneities were observed in terms of the beginning and end of the monthly activity of these species. The highest activity peaks of these species were in autumn as recorded in October in 2015, 2017 and 2018, and November in 2019 and 2020. *Aedes geniculatus* had a different seasonal activity pattern than *Ae. vexans* during the sampling years. The population of this species showed some secondary peaks in the first half of the sampling years (Fig. 5).

### Habitat Characteristics of *Ae. vexans* and *Ae. geniculatus*

As summarized in Table 4, the majority of *Ae. vexans* (87.77%) and *Aedes geniculatus* (98.9%) was observed in natural habitats. These *Aedes* species preferred to lay eggs in permanent and stagnant water in semi shady condition. *Aedes vexans* was found further in transparent waters (60.04%) with muddy floor (74.2%) and presence of plant out/surface and under water (61.78%), while *Ae. geniculatus* was observed in opaque waters without vegetation mainly in tree holes. Forest edge, Marsh, grassland were most preferred breeding sites of *Ae. vexans* in the province.

**Table 4:** Larval habitat characteristics of *Ae. vexans* and *Ae. geniculatus. *in Mazandaran Province, northern Iran during 2014-2020.

		*Ae. vexans*		*Ae. geniculatus*	
		N	%	N	%
Habitat status	Permanent	960	89.64	1276	100
	Temporary	111	10.36	0	0
Stream of water	Slow running	225	21.01	0	0
	Stagnant	846	78.99	1276	100
Status of vegetation	Without vegetation	420	39.22	1276	100
	With vegetation:			–	–
	Out of water	–	–	–	–
	Water surface	–	–	–	–
	Under	–	–	–	–
	All	651	60.78	–	–
Type of floor	muddy	795	74.2	1262	98.9
	Sandy	108	10.1	–	–
	Rocky	60	5.6	–	–
	Plastic	108	10.1	14	1.1
	Metal	–	–	–	–
Water status	Opaque	428	39.96	916	71.79
	Transparent	643	60.04	360	28.21
Light status	Sunny	214	19.98	–	–
	Semi shady	643	60.04	756	59.25
	Shady	214	19.98	520	40.75
Habitat type	Natural	940	87.77	1262	98.9
	Forest edge	70	7.45	–	–
	Marsh	420	44.68	–	–
	grassland	300	31.91	–	–
	Creek	0	0	–	–
	Pit	150	15.96	13	1.03
	Tree holes	–	–	1249	98.96
	Wetlands	–	–	–	–
	Springs	–	–	–	–
	Artificial	131	12.23	14	1.1
	Discarded tire	20	15.27	14	100
	pool	36	27.48	–	–
	Sewage	–	–	–	–
	Dam	–	–	–	–
	plastic dishes	–	–	–	–
	Tin dishes	–	–	–	–
	Concrete channel	–	–	–	–
	Pond	75	57.25	–	–

Adults of these species were more collected in forest sites as well as in the ports (Fig. 2). The forest sites were covered by dense and tall trees, meadows, shrubs and flowers. The cottages and other types of human dwellings in these sites were located at a distance of maximum 100 m apart with gable roofs and concrete, mud and wooden walls. The ports were covered (with grass and flowers and small shrubs.

The sampling sites also have administrative buildings, large sheds, buildings for employees to rest at a distance of approximately 1–50 m from each other with a gable roof and concrete, brick and iron walls.

### Mapping of historical and contemporary distribution records of *Aedes aegypti* and *Aedes albopictus* in Iran

**Figure 7:**
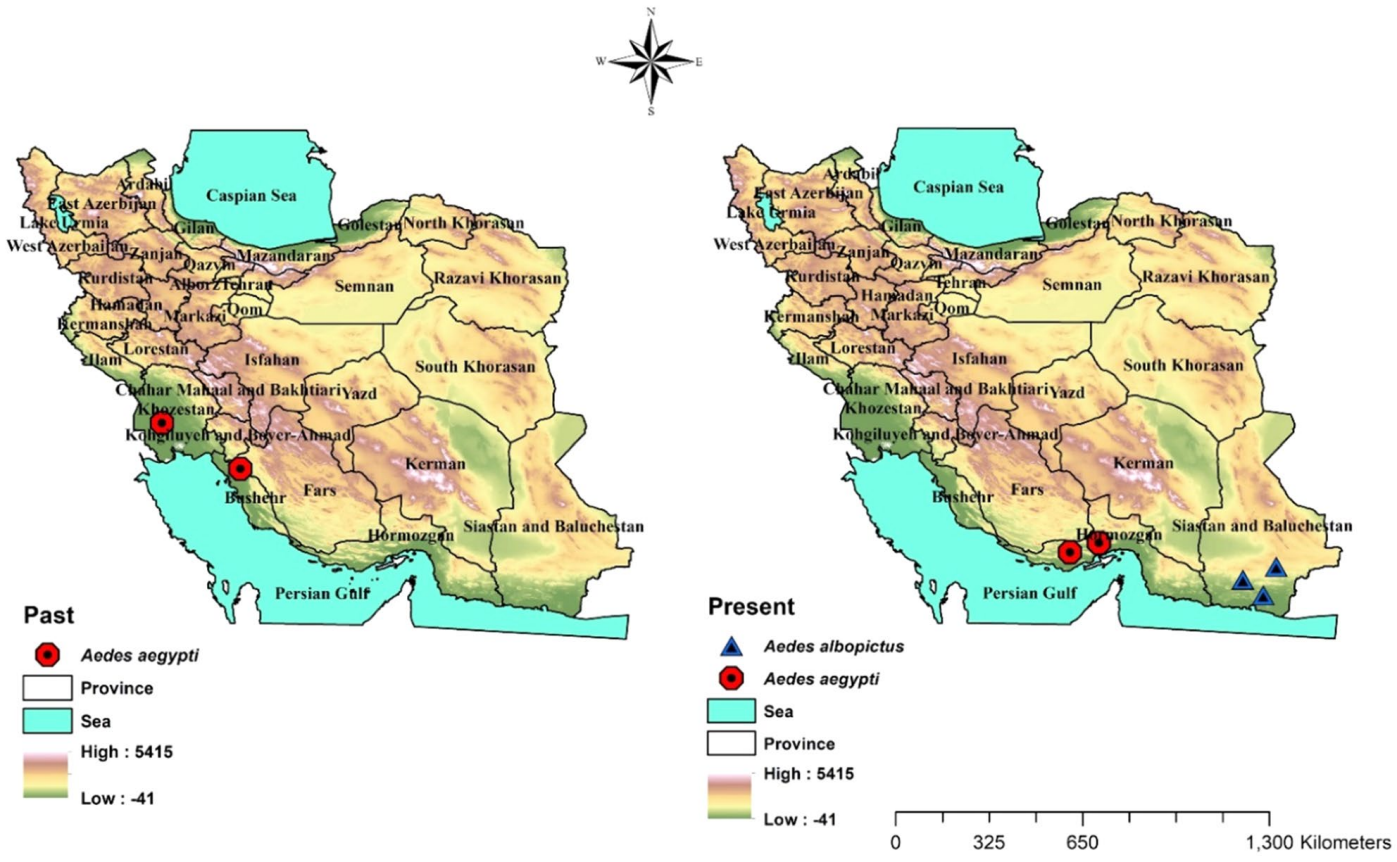
Historical (left side) and contemporary (right side) spatial distribution of *Ae. aegypti* and *Ae. albobictus* in Iran.

Figure 7 shows the spatial distribution of two species *Ae. aegypti* and *Ae. albopictus* in the past and present in Iran.

## Discussion

The present study provides the results of a comprehensive entomological surveillance program to assess the presence of invasive *Aedes* species in Mazandaran Province during 2014–2020. First and foremost, *Aedes aegypti* and *Ae. albopictus* the main vectors of dengue, chikungunya, Zika and yellow fever were not found at the points of entry and high-risk sites in Mazandaran Province throughout the surveillance period by none of the collection methods including ovitraps. Probably, one reason could be that these species have not yet entered or established in the province. Historically though, *Aedes aegypti* was active in the southern regions of Iran from 1920 to 1951^23–25^, it subsequently disappeared probably as a result of malaria eradication program which began in 1957^26^. Recently, *Ae. aegypti* has re-appeared in the ports of Khamir and Lengeh^11^ and more recently in Bandar Abbas (Iran Ministry of Health, personal communication) in Hormozgan Province, South of Iran. This revelation causes a national concern and as Bandar Abbas is an international and national trade hub, it warranted an intensified entomological surveillance throughout the country. *Aedes albopictus* was observed for the first time from the Sistan & Baluchestan Province bordering Pakistan in 2009, and in a coastal area near the county of Chabahar in the same province in 2013. Subsequently, intensive entomological surveillance failed to detect the establishment of the species^27^. However, surveillance and vigilance are emphasized throughout the country for successful planning and implementation of vector control programs^28,29^.

This was a top down surveillance strategy starting from the whole of the province down to the very exact counties with the potential points of entry (from outwards to inwards) to ascertain that *Ae. aegypti* and *Ae. albopictus* had not been established earlier in counties beyond the points of entry. Therefore, in the early years of the provincial surveillance program, the sampling process was carried out on a large scale throughout the province (16 counties). Subsequently, according to the national entomological surveillance protocol for *Aedes aegypti* and *Ae. albopictus*^11^, the surveillance program was limited to eight and then four counties that were considered high risk points of entry for the invasive *Aedes*. Specimens collected between May-December 2014 to 2020 provide baseline data on the relative abundance of *Aedes* species for the first time in the northern parts of Iran. The information builds up our understanding of the basic population dynamics, ecology and behaviour of local *Aedes* species.

In general, six local species were recorded and morphologically identified during the extensive surveillance program from 2014 to 2020. Based on the collected data, a map of the frequency distribution of the most abundant species of native *Aedes* in Mazandaran Province, northern Iran was drawn to provide up-to-date visual spatial distribution of the mosquitoes useful for control implementation if need be^30^ (Jemal and Al-Thukair 2018). *Aedes vexans* and *Ae. geniculatus* were collected more frequently in the study area, which is consistent with other studies in Iran^31–33^. To our Knowledge, there is limited and scattered data in the field of *Aedes* in Iran. In the studies conducted by Moradi Asal et al.^34^ three *Aedes* larvae i.e. *Ae caspius*, *Ae vexans* and *Ae. flavescens,* and by Paksa et al.^35^ two species of *Ae. caspius* and *Ae. vexans* were identified from Ardabil and East Azarbaijan Provinces, northwest of Iran, respectively. *Aedes geniculatus*, *Ae*. *echinus* and *Ae. caspius* were reported by Sofizade et al., in Kalaleh County, Golestan Province^36^. Moosa-Kazemi et al., also reported species of *Ae. vexans* and *Ae. caspius* from Kurdistan and Kermanshah Provinces^37^. It is worth noting that these studies are mostly highlighted all culicidae. In line with the national guidelines for prevention and control invasive *Aedes* species in the country^11^, five species of *Aedes* i.e. *Ae. vexans, Ae. caspius, Ae. caballus, Ae. flavescens, Ae. detritus* and *Ae. albopictus* were reported during Surveillance period from 2008 to 2014 in the provinces of Sistan & Baluchestan, Hormzgan, Bushehr, Kerman, Khuzestan and Korasan-e-Jonobi^38^.Recently, *Ae. vexans*, *Ae. geniculatus*, *Ae. echinus*, and *Ae. pulchritarsis* were also detected in higher priority entry points in Gilan Province, northern Iran^39^. So far, 12 species have been included in new Checklist of Iranian *Aedes*^40^*Aedes vexans*, known as the floodwater mosquito, is widely scattered in eastern Asia, North America, Western Africa, and much of Pacific Oceania^41^. The species is considered as the primary vector of Tahyna and Rift Valley fever, respectively^42,43^. Several other arborviruses have been isolated from this species around the world, including West Nile virus, Snowshoe hare virus, Jamestone Canyon virus and Batai virus^44^. Recently, Zika virus was detected in the salivary glands of the field-caught *Ae. vexans*^45^. West Nile virus is native to Iran and can be transmitted by the mosquitoes in the country. It has been detected in horses, birds and humans in 26 of the 31 Iranian provinces, especially in Caspian Sea littoral, northern Iran with vast wetlands^46–48^. Since *Ae. vexans* is considered as the competent vector of West Nile virus, also considering its mammalophilic and ornithophilic bahavior, it can strengthen the role of "bridge vector" between birds and humans^49^. Considering its high abundance in the study area, laboratory virus surveillance in its populations is recommended. Although *Ae. vexans* was not found infected with WNV in Mazandaran Province, the virus was detected in *Ae. caspius*^50^, a species with the third rank in terms of abundance in the present study^51^. Therefore, it shows the circulation of the virus in mosquito populations in the northern parts of Iran, and risk of entry and spread of arbovirus diseases in the country^52^.

Larvae of *Ae. geniculatus* were mainly observed in tree trunk cavities in northern part of Iran^33,36^. Similar to invasive *Aedes*, the species breeds in natural containers in woodland and man-made containers in the semi-urban and semi-domestic environments and adults coexist with *Ae. albopictus*^33,53,54^*.* It is a Palearctic mosquito species, dispersed in North Africa, the Middle East, and all over Europe^55,56^, and documented for the first time from Mazandaran Province, north of Iran^57^, followed by Ardebil, Golestan and Guilan Provinces^58,59^. In Vitro studies showed that *Ae. geniculatus* can transmit yellow fever, eastern equine encephalitis^60^, Dirofilaria immitis, repens^61^ and chikungunya virus^54^. Since the biology and ecology of *Ae*. *geniculatus* in Iran is poorly studied, further investigations are recommended.

*Aedes vexans* and *Ae. geniculatus* are known to be opportunistic feeders, day-active, exophilic mosquitoes that feed aggressively on birds, reptiles, humans and other mammals^56,62^. These species were collected with the highest mean frequency in Noshahr County (Fig. 3), a tourist destination and maritime trade hub in Mazandaran Province. Therefore, it poses a potential risk to human health in the area and highlights the importance of laboratory surveillance for arbovirus circulation in the region.

*Aedes flavescence* was found for the first time in the present study. The species was collected with low abundance in other parts of the world^15^. It was recorded for the first time in the form of larvae in West Azerbaijan in 1987 (Urmia city)^59^ and recently documented as a new species in Ardabil Province^34^. Based on the results of these studies and considering the detection of the species in the current study, it seems that the species is distributed in the northern parts of Iran. It should be noted that there is not much information on ecological aspects of the species in Iran, warranting further studies.

There are many resemblances and differences between mosquitoes in choosing of breeding sites, knowing the type of preferred larval habitat of mosquito species is very important in planning control measures at the right place and time and reducing resources^63^. In our study, the most abundant species i.e. *Ae. vexans* and *Ae. geniculatus* were found more in natural habitats, including swamps and tree holes, respectively, than in artificial habitats. These species were mostly collected from permanent, semi-shaded habitats with mud beds. In agreement with the present study, *Ae. geniculatus* was found in natural habitats without vegetation, with muddy water, permanent and slow-flowing water, muddy bed in Golestan province, northeastern Iran^36^. *Aedes vexans* was also observed in natural habitats with vegetation, clean water, muddy bed, permanent and stagnant water in Hormozgan Province, southern Iran^64^. It was reported that these species lay their eggs in habitats exposed to sunlight^36,65^, whereas, in the present study they prefer habitats with semi shady conditions. Moosa-Kazemi et al.^37^ reported that *Ae. vexans* tends to occupy habitats without vegetation in Kurdistan and Kermanshah Provinces, whereas our study and other studies^64,65^ showed that this species lays eggs in habitats with vegetation.

Unlike malaria vectors, there is not much data about the seasonal activity of *Aedes* species in Iran^66,67^. This is a preliminary report of monthly activities of the most abundant species of *Aedes* for the first time in northern Iran. In the present study, population fluctuations of *Ae. vexans* showed that its seasonal activity was mainly from May to December, while it was from June to December for *Ae. geniculatus*, both with the highest peaks in October and November. Wagner et al.^68^ reported that these species were more active in Autumn and are season-dependent species in most cases. *Aedes vexans* was reported to be active from June to September in the Aras Valley, Turkey^69^, and from May to August in Fars Province, southern Iran^70^. The largest peak of the species was recorded in June^71^, August^72^ and October^73^.

Climate and the environmental changes strongly affect the population dynamics of *Aedes* mosquitoes, alter the distribution, abundance, and longevity of mosquito species and consequently influences the epidemiology of vector-borne diseases worldwide^74^. Although comparison of monthly and yearly mean rainfall and the abundance of *Ae. vexans* and *Ae. geniculatus* populations in current study revealed some sort of correlation (in October and in Noshahr), this is not a consistent picture. Therefore, other variables such as physicochemical factors, vegetation, wind speed and predators may possibly be influencing the population variations of *Aedes* species in their habitats^75,76^. The complexity of the correlations between the rainfall and abundance of flood water mosquitoes e.g. *Ae. vexans* is shown by several studies^22,73,77,78^. Other meteorological factors including temperature and relative humidity may, on the other hand, play a role in defining the fluctuation of the populations of *Ae. vexans* and *Ae. geniculatus*^79^. These species were most frequently collected when and where the temperature and relative humidity are the highest. Also, no association was found between meteorological factors and other *Aedes* species, especially *Ae. caspius*, as the third abundant species in the present study. The population dynamics of *Ae. caspius* depends strongly on availability of areas flooded with brackish water during high tides^80^ as it tolerates high salinities owing to its high capacity for osmotic regulation^81^. The species was found in a wide variety of coastal sites, both fresh and saline marshes, but is most abundant in salt marshes, hence, its populations being less rainfall dependent than other species^80^.

Conclusion: This is the first comprehensive entomological surveillance in line with the national program in northern Iran that provides the basic information on *Aedes* species in the study area, the results of which can be useful for health decision makers in planning and implementing vector control programs in the future. *Aedes aegypti* and *Ae. albopictus* were not detected during the 7-year entomological surveillance in Mazandaran Province, northern Iran. However, since these species have recently been detected in southern Iran, Mazandaran Province and the whole of the country are going to be invaded sooner or later by these invasive *Aedes* species. This plus the fact that the most abundant species in the present study (*Ae. vexans* and *Ae. geniculatus*) are vectors of some pathogens, necessitates re-enforcing the national entomological surveillance program especially at high-risk areas such as airports, seaports, ground crossings and major routes for early detection of arrival of invasive species followed by prompt prevention and control programs.

## Material and methods

### Study area

The study was performed in Mazandaran Province in northern Iran. It has moderate and subtropical climate with average summer temperature of 25 °C and winter of about 8 °C. It lies between the southern coast of the Caspian Sea and central Alborz mountain range. The province has a population of 3,283,582 and an area of 23,842 km^2^. The latitude and longitude of the province are 36.5656^◦^ N and 53.0588^◦^ E. The province is bounded by Guilan Province in the West, Golestan Provinces in the East, Tehran and Semnan Provinces in the South and the Caspian Sea in the North. It has common trade borders with Russia in the North, the Republic of Azerbaijan in the West, and Turkmenistan and Kazakhstan in the East of the Caspian Sea. The Caspian Sea is located on the transport route of northern Europe and Asia with the south and is one of the axes of the north–south corridor. The Caspian Sea is also connected to open waters by the Volga River and the Volga-Don Canal. Trade through this canal may support the entry of invasive *Aedes*^19^.

### Study design

The study was planned in three parts in the province from 2014 to 2020, and presented in the form of a flowchart. Part “a” of the flowchart is management; including planning, coordination and organization, incorporating: (1) decision making by national and provincial health authorities; (2) intra- and inter-sectoral coordination and collaboration; (3) assessment of human resources and facilities; 4) theoretical and practical training; (5) provincial survey to determine the high risk points of entry and exact locations of sampling; (6) preparation of tools and equipment required for the monitoring program; and (7) drafting a sampling schedule. Part “b” of the flowchart is field studies including: (1) sampling; (2) transfer of specimens to the laboratory, and Part “c” of the flowchart is laboratory studies comprising of: (1) preparation of tools and solutions necessary for mounting and identification of species; and (2) sending a provincial report to the Ministry of Health. The final part of the flowchart is dedicated to the scenario in which *Ae. aegypti* and *Ae. albopictus* are found and the monitoring program will continue in the areas and focus on assessing the dynamics of the vector population, the virus in the field-collected *Aedes* vector and the design and planning of vector control measures. The whole procedure is followed by monitoring and evaluation as is outlined in the flowchart shown in Fig. 1.

### Mosquito collection and identification

The sampling followed a top down strategy i.e. from the whole of the counties of the province down to the very exact counties with point of entry in representative fixed sampling sites. In other words, to begin with and in the first year of the study, entomological surveillance was performed monthly from May to December 2014 in all of the 16 counties of the province. In 2015, the surveillance was performed bimonthly only in 8 counties with potential points of entry. From 2016 to 2020, the bimonthly surveillance was limited to counties with higher potential points of entries (ports and airports) in 4 counties throughout the province, as was suggested by the Iran CDC surveillance guideline of invasive *Aedes* vectors^11^. The names of the counties subjected to entomological surveillance are given in Table 1.

During the course of the entomological surveillance, four different sampling methods including ovitraps, larval collection, hand catch and human baited trap were employed. Ovitraps (100), containing 10% hay infusion, were installed bimonthly indoors and outdoors in selected points at each county. A total of 5400 ovitraps were placed in 54 selected sites during seven years of monitoring across the province (Fig. 2). They were visited for the presence of eggs 72 h later. Larval surveys were also conducted in the preferredartificial and natural breeding sites in a radius of 500 m from each point of entry (Fig. 2). Based on the shapes and sizes of the breeding sites, 350 cc dipper was used for larger, and pipette and dropper were used for smaller breeding sites. Water-holding containers on the deck of ships were also inspected for mosquito larvae. Fourth instar larvae were preserved in a glass of lactophenol solution and transferred to the laboratory, mounted on microscope slides using Berlese medium and identified morphologically using the key for the mosquitoes of Iran^20^. A total of 868 selected larval sampling points were visited during the 7-year monitoring program throughout the province. Larval habitat characteristics such as habitat type (natural or artificial), habitat condition (permanent or temporary, standing or flowing), vegetation type (with or without vegetation), floor type, water condition (clear or turbid) and condition Sunlight (full or partial light or shaded) were recorded. Human baited collections were performed fortnightly near breeding sites in 47 selected stations (Fig. 2) from morning to sunset using two human baits and one collector. The mosquitoes were pinned and identified using appropriate keys^20^. In addition, adult mosquitoes were collected with aspirator (hand catch) from various parts of ships, including bedrooms, kitchens, etc., arriving from Russia (Astrakhan and Makhachkala ports), Kazakhstan (Aktau port), Turkmenistan (Turkmenbashi port) and Azerbaijan (Baku port) to the ports of the Mazandaran Province.

A collection form was designed and used to record all collection data in the present study. Standard forms were used to report the surveillance data to the Ministry of Health.

### Collection of meteorological data

Meteorological information including temperature, rainfall and relative humidity were obtained from the Meteorological department of Mazandaran Province and used to analyze the relationship between these factors and the population fluctuation of *Aedes* species.

### Data analysis

Larvae and adults of each species were summed for statistical analyses and collectively reported in graphs and tables^2^. Samples were pooled for each habitat type regardless of collection date and reported as percentages^21^. Spearman’s test was used to assess the correlation between the *Aedes* population and the meteorological variables. A regression analysis model was performed to elucidate the relationship between *Aedes* populations and climatic factors. The mean (± standard deviation [SD]) number of *Aedes* species caught by month, year and county were computed and then compared using Kruskal–Wallis test followed by post hoc tests at a significance level of 5%. Statistical software SPSS ver. 25 was used to perform all the analyses^22^.

In order to perform the spatial analyses, geographical coordinates were extracted from 159 sampling sites (54 ovitrap, 58 larval collection, and 47 adult collection stations) using GPS software, entered into Excel in KML format, converted to “shape file”, and then transferred to ArcMap GIS10.8 software to prepare the spatial database of mosquitoes of Mazandaran Province, northern Iran.

### Ethical Statement

The study was designed according to the national ethical rules and regulations and approved by Ethic Committees of Mazandaran University of Medical Sciences (with Ethics codes: IR.MAZUMS.REC.1401.14363, IR.MAZUMS.REC.1397.353 and IR.MAZUMS.REC.1398.1020).

## Data Availability

The datasets generated and/or analyzed during the current study are presented in the manuscript and also are available from the corresponding author on reasonable request.
